# Computational Analysis of Damaging Single-Nucleotide Polymorphisms and Their Structural and Functional Impact on the Insulin Receptor

**DOI:** 10.1155/2016/2023803

**Published:** 2016-10-20

**Authors:** Zabed Mahmud, Syeda Umme Fahmida Malik, Jahed Ahmed, Abul Kalam Azad

**Affiliations:** ^1^Department of Genetic Engineering and Biotechnology, Shahjalal University of Science and Technology, Sylhet 3114, Bangladesh; ^2^Department of Biochemistry, North East Medical College Hospital, South Surma, Sylhet 3100, Bangladesh

## Abstract

Single-nucleotide polymorphisms (SNPs) associated with complex disorders can create, destroy, or modify protein coding sites. Single amino acid substitutions in the insulin receptor (INSR) are the most common forms of genetic variations that account for various diseases like Donohue syndrome or Leprechaunism, Rabson-Mendenhall syndrome, and type A insulin resistance. We analyzed the deleterious nonsynonymous SNPs (nsSNPs) in* INSR* gene based on different computational methods. Analysis of INSR was initiated with PROVEAN followed by PolyPhen and I-Mutant servers to investigate the effects of 57 nsSNPs retrieved from database of SNP (dbSNP). A total of 18 mutations that were found to exert damaging effects on the INSR protein structure and function were chosen for further analysis. Among these mutations, our computational analysis suggested that 13 nsSNPs decreased protein stability and might have resulted in loss of function. Therefore, the probability of their involvement in disease predisposition increases. In the lack of adequate prior reports on the possible deleterious effects of nsSNPs, we have systematically analyzed and characterized the functional variants in coding region that can alter the expression and function of* INSR* gene.* In silico* characterization of nsSNPs affecting* INSR* gene function can aid in better understanding of genetic differences in disease susceptibility.

## 1. Introduction

The insulin receptor (INSR) is a tyrosine kinase-specific transmembrane receptor that is activated by insulin, insulin growth factor I, and insulin growth factor II [1]. Metabolically, the INSR plays a crucial role in the regulation of glucose homeostasis which may result in a range of clinical events including diabetes and cancer [2, 3]. The main activity of INSR is persuading uptake of glucose and because of a decrease in insulin receptor signaling leads to diabetes mellitus type 2. The cells' inability to take glucose results in hyperglycemia and all the sequels that result in diabetes. Insulin-resistant patients may also display acanthosis nigricans. It is already proven that the presence of mutant receptors in the cell may have detrimental effects on the activity of the normal receptor. A previous study conducted with kinase-deficient INSRs transfected into cultured cells showed that such receptors suppressed the function of endogenous INSRs and functioned as dominant-negative mutations [4]. However, in most cases of insulin resistance, the mutation is expressed as a recessive form. Yamamoto-Honda et al. [5] studied the function and consequences of recessive mutation in the INSR. For example, Donohue syndrome known as Leprechaunism is a rare and severe genetic autosomal recessive disorder due to defect in the* INSR* gene.

Single-nucleotide polymorphisms (SNPs) are the most common form of human genetic variations and nearly half a million of SNPs reside in the exons of the human genome. Among these SNPs, “nonsynonymous SNPs (nsSNPs)” can alter the amino acid residues and contribute to functional diversity in encoded proteins in the human population. The genomic distribution of SNPs is not obviously homogenous. In general, SNPs occur in noncoding regions more frequently than in coding regions [6]. Genetic recombination and mutation rate are some other factors that can also determine SNP density [7]. SNPs are usually biallelic and a single SNP may cause a Mendelian disease [8]. In case of complex diseases, SNPs do not usually function independently but rather they work as a group with other SNPs to exhibit a disease condition which has been seen in osteoporosis [9]. A wide range of human diseases, such as sickle-cell anemia, *β* thalassemia, and cystic fibrosis, result from SNPs [10–12]. For drug discovery, diseases with different SNPs may become crucial pharmacogenomic targets; some SNPs are also associated with the metabolism of different drugs [13–15]. For genome-wide association studies, SNPs can serve as a useful genetic marker [16]. The consequences or deleterious effects of SNPs are generally attributed to their impact on the protein structure and function. However, very few studies have been done to predict the SNPs and their impacts on INSR.

In this study, we identified the nsSNPs' deleterious mutations* in silico* which may have an impact on the structural integrity of human INSR protein and are involved in several genetic diseases. Knowledge of* in silico* analysis of SNPs will play a major role in the understanding of the genetic basis of several complex genetic human diseases. Furthermore, the genetics of human phenotypic diversity could also be implied by establishing the functions of these SNPs. Using laboratory techniques, it is still a major obstacle to identify the functional SNPs in a disease-related gene. However, with recent advancements in the* “in silico”* technique and procedures, it is now possible to carry out research investigations without the need for extensive lab work. The main focus of this work is to investigate the SNPs genetic variations in the human* INSR* gene and their possible effects on structure and functions of INSR using bioinformatics and computational algorithms.

## 2. Materials and Methods

### 2.1. Datasets

The data of human* INSR* gene was collected from Online Mendelian Inheritance in Man (OMIM) and Entrez Gene on National Center for Biological Information (NCBI) web sites. The SNPs information (protein accession number and SNP ID) of the* INSR* gene was retrieved from the NCBI dbSNP (http://www.ncbi.nlm.nih.gov/snp/).

### 2.2. Analysis of Protein Variation Effects

A sequence based predictor estimates the effect of protein sequence variation on protein function. Many web servers are available to predict the effect of single amino acid variations on protein stability and protein binding efficiency. PROVEAN (http://provean.jcvi.org/index.php), I-Mutant 3.0 (http://gpcr2.biocomp.unibo.it/cgi/predictors/I-Mutant3.0/I-Mutant3.0.cgi), and PolyPhen (http://genetics.bwh.harvard.edu/pph2/) were used in this study.

In PROVEAN, protein sequences of BLAST hits with more than 75% global sequence identity were clustered together and top clusters formed a supporting sequence set. A delta alignment scoring system was used, where the scores of each supporting sequence were averaged within and across clusters to generate the final PROVEAN score. A protein variant is said to be “deleterious” if the final score is below a certain threshold (default is −2.5) or is predicted to be “neutral” if the score is above the threshold [17].

PolyPhen version 2 predicts the influence of amino acid substitution on the structure and function of proteins by using the specific empirical rules. Protein sequence, database ID/accession number, amino acid position, and amino acid variant details are the input options for PolyPhen [18]. The tool estimates the position-specific independent count (PSIC) score for every variant and calculates the score difference between variants.

I-Mutant 2.0 and I-Mutant 3.0 are based on Support Vector Machine (SVM) algorithm to predict the stability of the protein due to single amino acid variations. It can predict protein stability changes by using protein sequence or structure. It has an overall accuracy of 77% when prediction is based on protein sequence. I-Mutant 2.0 and I-Mutant 3.0 predict the DDG values as a regression estimator and the sign of the stability change. I-Mutant 3.0 furthermore classifies mutations into three categories: neutral mutation (−0.5 ≤ DDG ≤ 0.5), large decrease (≤ −0.5), and large increase (>0.5) [19, 20].

### 2.3. 3D Modeling and Analysis of Protein Structure

The EMBL-EBI web-based tool PDBsum (http://www.ebi.ac.uk/pdbsum/) was used to find the proteins related to the INSR. PDBsum provides an at-a-glance overview of every macromolecular structure deposited in the Protein Data Bank (PDB). It performs a FASTA search against all sequences in the PDB to obtain a list of the closest matches [21]. LS-SNP/PDB [22] annotates all human SNPs that produce an amino acid change in a protein structure in PDB [23], using features of their local structural environment, putative binding interactions, and evolutionary conservation. The presence of an nsSNP in a highly conserved surface patch or a charged surface patch suggests possible biological importance. These annotations allow users to quickly scan a large number of nsSNPs of interest and prioritize those with higher likelihood of impacting normal protein activities. LS-SNP server is also useful to analyze human nsSNPs onto protein homology models [24].

PYMOL was used to generate the mutant models of each of the selected PDB entries for the corresponding amino acid substitutions. PYMOL allows browsing through a rotamer library to change amino acids. A “Mutagenesis Wizard” was used to replace the native amino acid with new one. The mutation tool facilitates the replacement of the native amino acid by the “best” rotamer of the new amino acid. The “.pdb” files were saved for all the models.

### 2.4. Structure Validation and Energy Minimization

Structural Analysis and Verification Server (SAVES) was implemented for evaluating the quality and validation of the refined 3D structural models. The SAVES integrates PROCHECK, PROVE, and ERRAT software programs to check overall quality of the 3D models obtained from the PYMOL mutagenesis tool. Structure refinement was carried out using KoBaMIN which is based on knowledge based potential refinement for proteins protocol [25].

### 2.5. Protein Stability Validation for Mutant Structure

The approach called Mutation Cutoff Scanning Matrix (mCSM) uses the concept of graph-based structural signatures to study and predict the impact of single-point mutations on protein stability and protein-protein and protein-nucleic acid affinity. The mCSM encodes distance patterns between atoms to represent protein residue environments [26].

### 2.6. Structural Analysis

**Figure 1:**
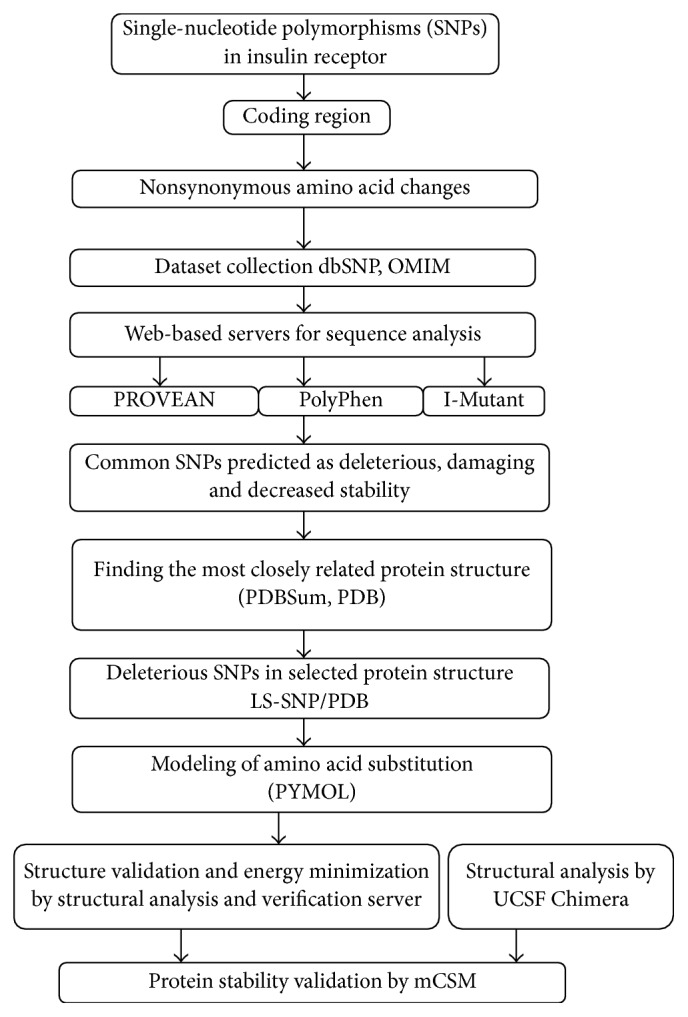
Workflow of* in silico* approaches used in this study.

The predicted structures were viewed in University of California San Francisco (UCSF) Chimera. It is a computationally intensive program for visualization of molecular models and it provides an interactive interface for the user for analyzing the models and model related data. It provides a platform for analyzing sequence alignments, generating homology models, molecular docking, viewing various density models, and also comparing different models by superimposition [27]. The mutant and wild type structures were superimposed and the effect of the nonsynonymous variation was observed in terms of steric hindrance due to the changes of the side chains and charge of the amino acid. Then, the degree of change in the hydrophobicity or hydrophilicity of the substituted amino acid and its effect on the interacting intrachain and interchain molecules was analyzed. A summary of* in silico* approaches used in this study is shown in Figure 1.

## 3. Results

### 3.1. SNP Dataset from dbSNP

The dbSNP contains both validated and nonvalidated polymorphisms. In spite of this drawback, we opted to avail the dbSNP because allelic frequency of most of nsSNPs of INSR has been recorded there and that is the most extensive SNP database. In our data search, some previously reported SNPs in dbSNP have been identified as invalid because of wrong sequencing and alignment. These erroneous SNPs have expired or have merged with other SNPs. Some* INSR* genes have been renamed. We carefully cross-examined the databases and removed those old and invalid SNPs. At dbSNP,* INSR* gene contains data for 4967 SNPs. Out of 4967 SNPs, only 57 were nsSNPs in the coding region (Table 1). Our investigation accounted for the nsSNPs in the coding region only.

### 3.2. Effects of nsSNPs on INSR Predicted by Different Tools

The PROVEAN algorithm works mainly with primary sequence for prediction while other tools perform similar task with the structure. Since PROVEAN can predict a large number of substitutions and does not require structures, it is advantageous over other tools. PROVEAN predicts the effect of the variant on the biological function of the protein based on sequence homology. The scores of PROVEAN are classified as “deleterious” below a certain threshold (here −2.5) and “neutral” above it. A  .txt file containing “db SNP rsIDs” of all 57 nsSNPs was submitted to the “dbSNP rsIDs” page to calculate the PROVEAN score. Out of 57 nsSNPs, PROVEAN predicted 24 as deleterious and 33 as neutral (Table 1). Among the 24 deleterious nsSNPs mutations, W1220L and C219R were predicted as highly deleterious with PROVEAN scores of −11.648 and −9.831, respectively.

PolyPhen identifies homologues of the input sequences via BLAST and calculates PSIC scores for every variant and estimates the difference between the variant scores; the difference of 0.339 is detrimental. There are certain empirical rules applied to the sequences and the accuracy is approximately 82% with a chance of 8% false-positive prediction. The protein accession number of INSR (P06213) and the amino acid substitutions corresponding to each of the 57 nsSNPs were submitted separately.  Table 2 summarizes the results obtained from the PolyPhen server. A PSIC score difference was assigned to categorize SNPs as benign and damaging. “PolyPhen-2: scores are evaluated as 0.000 (most probably benign) to 0.999 (most probably damaging).” Twenty-one of the 57 nsSNPs were predicted as “damaging,” and the PSIC scores fell into the range of 1.51 to 3.41. 18 nsSNPs predicted to be deleterious by the SIFT (Sorting Intolerant from Tolerant) program were also predicted to be damaging by the PolyPhen server.

I-Mutant is a neural network based routine tool used in the analysis of protein stability alterations by considering the single-site mutation. I-Mutant also provides the scores for free energy alterations, calculated with the FOLD-X energy based web server. By assimilating the FOLD-X estimations with those of I-Mutant, about 93% precision can be achieved. We have considered a threshold of −1.5 Kcal/mol to predict a SNP to be destabilized. Forty-six nsSNPs were considered as destabilized with DDG values by I-Mutant (Table 3). Finally, we selected 18 significant nsSNPs because they were predicted to be deleterious by PROVEAN, PolyPhen, and SIFT programs and showed decreased structural stability following analysis by I-Mutant (Table 4).

### 3.3. Effects of nsSNPs on Protein Structure

By using the EMBL-EBI web-based tool PDBsum, the INSR protein structures were searched. Two related protein structures, namely, 2HR7 and 4IBM, were found to share 100% amino acid sequence similarity. The single amino acid polymorphism (SAAP) database server (http://www.bioinf.org.uk/saap/db/) is offline due to essential maintenance. Thus, we were unable to map the deleterious nsSNPs into protein structure through SAAP. Mapping the deleterious nsSNPs into protein structure information was performed through the LS-SNP/PDB server.

According to this resource, 2HR7 accounted for 9 nsSNPs and 4IBM had 4 nsSNPs. Apart from the SNP scanning, LS-SNP/PDB server also predicts solvent accessibility and conservation ratio of given protein structures. An overview of mapping of mutant structures and their solvent accessibility and conservation ratios is given in Table 5.

**Figure 2:**
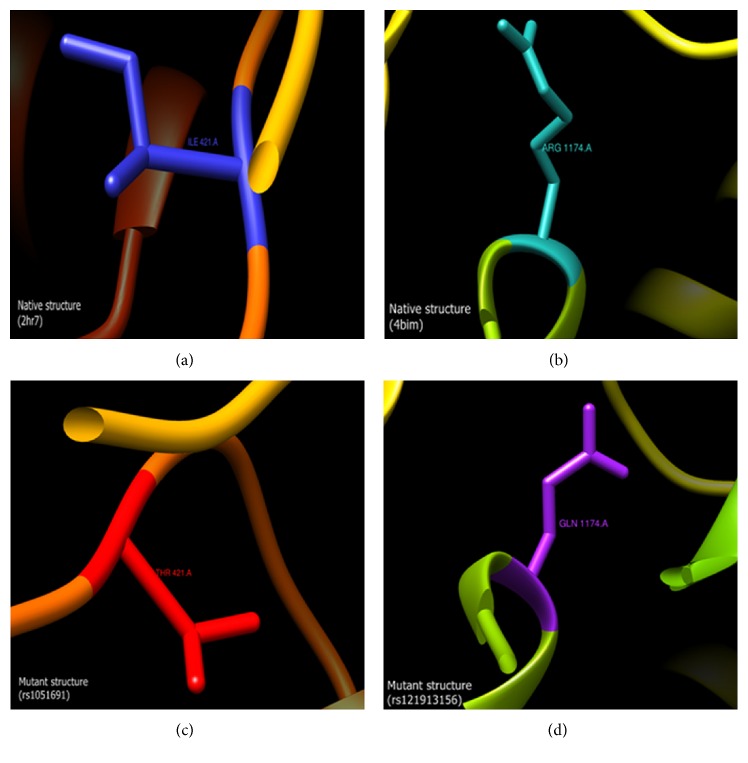
A comparison of amino acid substitutions due to nsSNPs. Two mutant structures of deleterious nsSNPs rs1051691 (I421T) (c) and rs121913156 (R1174Q) (d) were compared to their native structures 2HR7 (a) and 4IBM (b), respectively. Models were generated by using PYMOL and visualized by UCSF Chimera.

Out of 18 nsSNPs predicted to be deleterious by PROVEAN or PolyPhen, a total of 13 were mapped to the PDB ID 2HR7 and 4IBM native structures. All the functional nsSNPs predicted using the PROVEAN and PolyPhen tools were subjected to the PYMOL mutation tool. A model for each functional nsSNP was made by PYMOL mutagenesis tool and visualized using UCSF Chimera tool for comparison with the native structures (Figure 2, only mutants rs1051691 (I421T) and rs121913156 (R1174Q) are shown).

**Figure 3:**
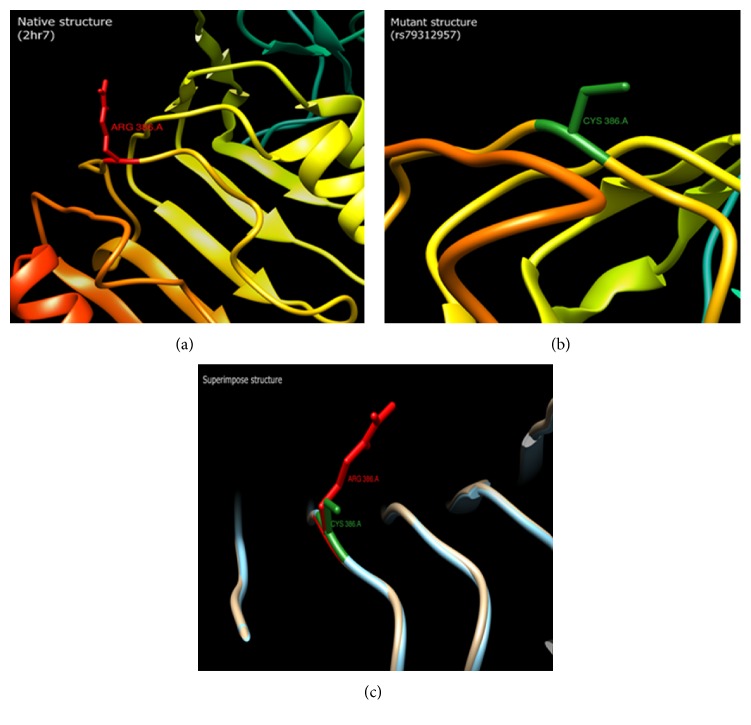
Superimposition of native and mutant structures. Native structure (2HR7) shows arginine at position 386 (a) and mutant modeled structure rs79312957 (R386C) shows cysteine residue at the corresponding position (b). (c) shows the superimposition of the native structure (red) with mutant modeled structure (green) in position 386.

Energy minimization is performed for the native structures (2HR7 and 4IBM) and the mutant modeled structures. The KoBaMIN web server uses a force field for energy minimization. The total energy for all the mutant and native models after minimization is listed in Table 6. The total energies for the native structures of 2HR7 and 4IBM are −22087.6969 kJ/mol and −13041.4646 kJ/mol, respectively. Change in total energy due to mutation is noticeable in the both 2HR7 and 4IBM mutant models. RMSD is the measure of the deviation of the mutant structures from their native configurations. The higher the RMSD value, the more the deviation between the two structures. Structural changes, in turn, affect functional activity. RMSDs for all the mutant structures are listed in Table 6. The mutants rs79312957 and rs121913156 have higher RMSD value of 6.025 and 0.436 compared to native structures RMSD value 6.019 and 0.404, respectively. These two nsSNPs could be believed to affect the structure of the proteins. These two nsSNPs were also shown to be deleterious according to the PROVEAN and PolyPhen server. The 3D structure of the native INSR protein crystal structures 2HR7 and 4IBM and the predicted mutant structures were superimposed over chain A. The superimposed structures revealed that the mutants might have considerably affected the protein structure and thus its function (Figure 3; only rs79312957 is shown). Substituted amino acid residues in the mutants might have altered the conformation of the INSR or networking among neighboring amino acids or interaction between the substrate and receptor [28, 29].

### 3.4. Effects of nsSNP on Protein Stability

The effects of the nsSNPs on protein stability were computed with FOLD-X by mCSM server which uses an empirical energy equation to calculate the Gibbs free energy DDG. The empirical energy terms consider the location and type of a substituted residue. The mCSM is a structure based prediction tool. Two different analysis protocols were utilized to obtain maximum information over the effect of the single amino acid substitutions: (1) all the nsSNPs were considered singularly and their effect on the protein stability and interaction potential was determined; (2) the nsSNPs were considered according to the allelic sequences. Initially, all the structures were minimized and obtained a stable protein stability value. Then the structures for each single amino acid variation were generated using the Build Model feature of FOLD-X 3.0. Finally, the effect of each single amino acid variation on the protein stability of INSR was determined using the analyzed complex features. The mutation was considered as destabilizing and stabilizing when the DDG was >0 and <0, respectively. In this prediction method, all the mutant structures ultimately derived from the PROVEAN, PolyPhen, and I-Mutant programs were finally submitted to the mCSM server to predict mutant structure's protein stability upon mutation. The mCSM predicted all structures as “Destabilizing” including two as “Highly Destabilizing” (Table 7).

## 4. Discussion

The SNP in* INSR* can manifest several insulin-resistant syndromes like Leprechaunism, Rabson-Mendenhall syndrome, and type A insulin resistance [30, 31]. Diagnostic measures have already been established on clinical examination as well as laboratory diagnostic tests with elevated insulin levels as a constant feature. Functional and DNA analysis can be used for absolute confirmation, but certain mutations do not contribute to insulin binding and DNA analysis is still not able to identify all the putative mutations. Although there is no direct genotype-phenotype correlation, but mutations in the alpha subunit of the insulin receptor are associated with a more severe phenotype compared to the mutations affecting the beta subunit [32]. Numerous studies have been conducted using* in silico* analysis approaches to predict the functional effects of nsSNPs on genes such as* G6PD*,* BARF,* and* PTEN* [33, 34]. Therefore, for addressing this issue, we selected* in silico* strategy to analyze and predict the functional effects of SNPs on INSR.

We used different* in silico* methods based on the combination of two distinctive approaches which are sequence and structural based approaches. In comparison with the structure based methods, sequence based prediction methods are one step ahead because they can be applied to any proteins with known relatives, whereas structure based approaches are not feasible to implement for proteins with unknown 3D structures. Software programs and servers that integrate both sequence and structure resources have advantage of being able to assess the authenticity of the predicted results by cross-referencing the results from both methods. Most computational methods utilize this information for the prediction and analyses of deleterious nsSNPs, among which PROVEAN and PolyPhen algorithms are the main representatives. Considering normalized probability score below −2.5 in PROVEAN and a PSIC score 1.5 in PolyPhen as deleterious, 24 and 21 of amino acid substitutions were predicted to have functional impact on INSR gene. The variation in prediction score of PROVEAN and PolyPhen is mainly because of the difference in sequence alignment and the values used to classify the variants. Significant similarity was observed between the results obtained by PROVEAN and PolyPhen. PROVEAN and PolyPhen in predicting the effect of nsSNPs on protein function might be suitable* in silico* approach [35].

In order to predict the impact of nsSNPs on protein structure, I-Mutant 3.0 was used which evaluated the stability change upon single-site mutation. I-Mutant 3.0 was ranked as one of the most reliable predictors based on the work performed by Khan and Vihinen [36]. Based on the difference in Gibbs free energy value of mutant and wild type protein, 45 nsSNPs were found to largely destabilize the protein. Structures of the several human INSRs are available in PDB and have been used to analyze the effect of polymorphisms. A 3D structure is essential for analyzing the impact of the SNPs in structural level. Therefore, we predicted the 3D structure most similar to human INSR through the EMBL PDBsum program. Depending on the highest sequence similarity and alignment, we selected 2HR7 and 4IBM from the PDB. Already predicted deleterious and disease-related nsSNPs predicted by PROVEAN and PolyPhen were further subjected to LS-SNP/PDB server for mapping SNPs in 2HR7 and 4IBM crystal structures. The mutant structures served as valuable tool to compare and predict protein stability, RMSD, and energy calculation between wild type and mutant type structures.

Each mutation was considered individually to study the inherent effect of the SNP. In addition, the allelic sequences were analyzed to investigate if the polymorphisms neutralized each other by occurring simultaneously as an act of preservation of function by nature. The mCSM was used to analyze the effects of single amino acid variations on the structure and stability of the protein.

Our results indicated that all of the 13 mutant structures of 2HR7 and 4IBM were predicted as “Destabilizing” which signified our results found by PROVEAN and PolyPhen. Among all the destabilized mutant structures, two mutants were labelled as “Highly Destabilizing” which were rs1051691 and rs52800171 in their I448T and W1220L positions, respectively, which suggested that these polymorphisms should be considered as a potential target for future experiments. If a single amino acid variation shows a change in protein stability or protein-protein interaction, it should give comparable values with the sign reversal for the reverse mutation. This would indicate that the prediction of the effect of the single amino acid variation on the protein structure or protein-protein interaction might be substantial.

## 5. Conclusions

This study shows a correlation between SNPs in the* INSR* gene and several diseases like insulin-resistant syndromes such as Leprechaunism, Rabson-Mendenhall syndrome, and type A insulin resistance. The present study concludes that 13 nsSNPs especially rs1051691 and rs52800171 decreases protein stability and are not tolerated or may result in loss of function. Their presence in the INSR increases the possibility of altered transcriptional and cell cycle regulation and INSR mediated diseases. Therefore, the probability of their involvement in disease predisposition increases. Thus, for further analysis, these mutations should be given priority to obtain detailed information on their effects. In order to confirm the structures modeled in this study, the actual structures should be determined by X-ray crystallography or nuclear magnetic resonance spectroscopy. We anticipate that the results obtained from our analysis would pave the way for providing useful information to the researchers and can play an important role in bridging the gap between biologists and bioinformaticians.

## Figures and Tables

**Table 1:** List of nsSNPs that were predicted by PROVEAN to have functional significance.

SNP_ID	Mutation	PROVEAN results	PROVEAN score
rs1799816	V1012M	Neutral	−2.418
rs52836744	G58R	Deleterious	−6.762
rs121913144	R1027^*∗*^		
rs121913145	H236R	Deleterious	−5.616
rs121913156	R1201Q	Deleterious	−3.701
rs891087	D261E	Neutral	−0.116
rs2162771	P830L	Neutral	−1.576
rs13306449	Y1361C	Deleterious	−4.157
rs35045353	G811S	Neutral	−2.401
rs1051691	I448T	Deleterious	−3.774
rs1051692	Y171H	Neutral	−1.304
rs2229429	D546E	Neutral	−2.474
rs7508518	A2G	Neutral	0.465
rs52800171	W1220L	Deleterious	−11.648
rs55816055	S353P	Deleterious	−2.761
rs56395521	L1065V	Neutral	−1.133
rs72549237	V362I	Neutral	−0.211
rs76077021	R889W	Deleterious	−4.254
rs76673783	E664G	Deleterious	−5.068
rs78433961	R796S	Neutral	−0.984
rs78827745	M65K	Deleterious	−3.808
rs79312957	R413C	Deleterious	−5.623
rs113527718	S1297G	Deleterious	−2.539
rs138528064	T320M	Neutral	−1.959
rs140762552	T107M	Deleterious	−2.922
rs140852238	E51K	Deleterious	−2.545
rs141484557	G262S	Deleterious	−3.037
rs142391704	A706D	Neutral	0.622
rs142910337	D75G	Neutral	1.672
rs143523271	S748L	Neutral	−0.793
rs143919163	G192D	Neutral	−1.884
rs144029037	V900I	Neutral	−0.698
rs146588336	D946E	Neutral	−0.743
rs147671523	E517G	Deleterious	−4.293
rs148838377	P755S	Neutral	−0.076
rs149536206	H8598^*∗*^		
rs150114699	L991I	Neutral	−1.71
rs181150880	R410Q	Neutral	−2.011
rs182552223	T858A	Deleterious	−2.612
rs183360558	D893N	Neutral	−1.752
rs185736681	R1053C	Deleterious	−5.933
rs187282966	R889Q	Neutral	−1.492
rs199580495	S1033F	Deleterious	−5.642
rs199599404	M1319I	Neutral	−1.196
rs199659271	C219R	Deleterious	−9.831
rs200059069	K411Q	Neutral	−1.21
rs200110540	V866I	Neutral	−0.134
rs200199169	P271L	Neutral	−2.315
rs200400127	A1340V	Neutral	−1.519
rs200921389	G1048D	Neutral	−1.585
rs201147780	K294R	Neutral	−0.904
rs201466857	T858M	Deleterious	−3.384
rs201506342	P1312T	Deleterious	−3.008
rs201978448	A537V	Deleterious	−3.252
rs201979105	S1221A	Deleterious	−2.698
rs202160383	R1128H	Neutral	−2.063

^*∗*^Premature stop codon.

**Table 2:** Potential effect of amino acid substitution for nsSNPs in human INSR predicted by the PolyPhen algorithm.

SNP_ID	Mutation	PolyPhen results	Score	Sensitivity	Specificity
rs1799816	V1012M	Probably damaging	0.992	0.7	0.97
rs52836744	G58R	Probably damaging	1	0	1
rs121913144	R1027^*∗*^				
rs121913145	H236R	Probably damaging	1	0	1
rs121913156	R1201Q	Probably damaging	1	0	1
rs891087	D261E	Benign	0	1	0
rs2162771	P830L	Benign	0	1	0
rs13306449	Y1361C	Probably damaging	1	0	1
rs35045353	G811S	Benign	0.441	0.89	0.9
rs1051691	I448T	Probably damaging	0.996	0.55	0.98
rs1051692	Y171H	Benign	0.024	0.95	0.81
rs2229429	D546E	Benign	0.032	0.95	0.82
rs7508518	A2G	Benign	0	1	0
rs52800171	W1220L	Probably damaging	1	0	1
rs55816055	S353P	Possibly damaging	0.528	0.88	0.9
rs56395521	L1065V	Benign	0	1	0
rs72549237	V362I	Benign	0.003	0.98	0.44
rs76077021	R889W	Benign	0.111	0.93	0.86
rs76673783	E664G	Possibly damaging	0.592	0.87	0.91
rs78433961	R796S	Benign	0.001	0.99	0.15
rs78827745	M65K	Possibly damaging	0.934	0.8	0.94
rs79312957	R413C	Probably damaging	0.999	0.14	0.99
rs113527718	S1297G	Benign	0.004	0.97	0.59
rs138528064	T320M	Benign	0.199	0.92	0.88
rs140762552	T107M	Probably damaging	1	0	1
rs140852238	E51K	Benign	0.003	0.98	0.44
rs141484557	G262S	Possibly damaging	0.939	0.8	0.94
rs142391704	A706D	Benign	0	1	0
rs142910337	D75G	Benign	0	1	0
rs143523271	S748L	Benign	0	1	0
rs143919163	G192D	Benign	0.005	0.97	0.74
rs144029037	V900I	Benign	0.048	0.94	0.83
rs146588336	D946E				
rs147671523	E517G	Possibly damaging	0.726	0.86	0.92
rs148838377	P755S	Benign	0	1	0
rs149536206	H8598^*∗*^				
rs150114699	L991I	Benign	0.442	0.89	0.9
rs181150880	R410Q	Possibly damaging	0.935	0.8	0.94
rs182552223	T858A	Benign	0.007	0.96	0.75
rs183360558	D893N	Benign	0	1	0
rs185736681	R1053C	Benign	0.223	0.91	0.88
rs187282966	R889Q	Benign	0.252	0.91	0.88
rs199580495	S1033F	Probably damaging	0.968	0.77	0.95
rs199599404	M1319I	Benign	0.007	0.96	0.75
rs199659271	C219R	Probably damaging	1	0	1
rs200059069	K411Q	Possibly damaging	0.75	0.85	0.92
rs200110540	V866I	Benign	0	1	0
rs200199169	P271L	Benign	0	1	0
rs200400127	A1340V	Benign	0.021	0.95	0.8
rs200921389	G1048D	Benign	0.009	0.96	0.77
rs201147780	K294R	Benign	0.008	0.96	0.76
rs201466857	T858M	Probably damaging	0.994	0.69	0.97
rs201506342	P1312T	Possibly damaging	0.616	0.87	0.91
rs201978448	A537V	Benign	0.143	0.92	0.86
rs201979105	S1221A	Probably damaging	0.997	0.41	0.98
rs202160383	R1128H	Benign	0.031	0.95	0.82

^*∗*^Premature stop codon.

**Table 3:** List of nsSNPs' stability predicted by I-MUTANT.

SNP_ID	Mutation	Stability	SNP_ID	Mutation	Stability
rs1799816	V1012M	Decrease	rs141484557	G262S	Decrease
rs52836744	G58R	Decrease	rs142391704	A706D	Decrease
rs121913144	R1027^*∗*^		rs142910337	D75G	Decrease
rs121913145	H236R	Decrease	rs143523271	S748L	Increase
rs121913156	R1201Q	Decrease	rs143919163	G192D	Decrease
rs891087	D261E	Increase	rs144029037	V900I	Decrease
rs2162771	P830L	Decrease	rs146588336	D946E	Increase
rs13306449	Y1361C	Increase	rs147671523	E517G	Increase
rs35045353	G811S	Decrease	rs148838377	P755S	Decrease
rs1051691	I448T	Decrease	rs149536206	H8598^*∗*^	
rs1051692	Y171H	Decrease	rs150114699	L991I	Decrease
rs2229429	D546E	Increase	rs181150880	R410Q	Decrease
rs7508518	A2G	Decrease	rs182552223	T858A	Decrease
rs52800171	W1220L	Decrease	rs183360558	D893N	Decrease
rs55816055	S353P	Increase	rs185736681	R1053C	Decrease
rs56395521	L1065V	Decrease	rs187282966	R889Q	Decrease
rs72549237	V362I	Decrease	rs199580495	S1033F	Increase
rs76077021	R889W	Decrease	rs199599404	M1319I	Decrease
rs76673783	E664G	Decrease	rs199659271	C219R	Decrease
rs78433961	R796S	Decrease	rs200059069	K411Q	Increase
rs78827745	M65K	Decrease	rs200110540	V866I	Decrease
rs79312957	R413C	Decrease	rs200199169	P271L	Decrease
rs113527718	S1297G	Decrease	rs200400127	A1340V	Decrease
rs138528064	T320M	Decrease	rs200921389	G1048D	Decrease
rs140762552	T107M	Decrease	rs201147780	K294R	Increase
rs140852238	E51K	Decrease	rs201466857	T858M	Decrease
rs201506342	P1312T	Decrease	rs201979105	S1221A	Decrease
rs201978448	A537V	Increase	rs202160383	R1128H	Decrease

^*∗*^Premature stop codon.

**Table 4:** Common amino acid change due to deleterious nsSNPs in human INSR predicted by PROVEAN and PolyPhen algorithms.

SNP_ID	Mutation	PROVEAN results	PROVEAN score	PolyPhen results	PolyPhen score
rs52836744	G58R	Deleterious	−6.762	Probably damaging	1
rs121913145	H236R	Deleterious	−5.616	Probably damaging	1
rs121913156	R1201Q	Deleterious	−3.701	Probably damaging	1
rs13306449	Y1361C	Deleterious	−4.157	Probably damaging	1
rs1051691	I448T	Deleterious	−3.774	Probably damaging	0.996
rs52800171	W1220L	Deleterious	−11.648	Probably damaging	1
rs55816055	S353P	Deleterious	−2.761	Possibly damaging	0.528
rs76673783	E664G	Deleterious	−5.068	Possibly damaging	0.592
rs78827745	M65K	Deleterious	−3.808	Possibly damaging	0.934
rs79312957	R413C	Deleterious	−5.623	Probably damaging	0.999
rs140762552	T107M	Deleterious	−2.922	Probably damaging	1
rs141484557	G262S	Deleterious	−3.037	Possibly damaging	0.939
rs147671523	E517G	Deleterious	−4.293	Possibly damaging	0.726
rs199580495	S1033F	Deleterious	−5.642	Probably damaging	0.968
rs199659271	C219R	Deleterious	−9.831	Probably damaging	1
rs201466857	T858M	Deleterious	−3.384	Probably damaging	0.994
rs201506342	P1312T	Deleterious	−3.008	Possibly damaging	0.616
rs201979105	S1221A	Deleterious	−2.698	Probably damaging	0.997

**Table 5:** Mapping of nsSNPs in 2HR7 and 4IBM 3D structures.

SNP_ID	Mutation	PDB residue number	Solvent accessibility	Conservation
*2HR7*				
rs52836744	G58R	31	Intermediate 10%	5%
rs121913145	H236R	209	Intermediate 23%	10%
rs1051691	I448T	421	Buried 1%	1%
rs55816055	S353P	326	Buried 5%	3%
rs78827745	M65K	38	Buried 0%	8%
rs79312957	R413C	386	Exposed 58%	3%
rs140762552	T107M	80	Buried 2%	5%
rs141484557	G262S	235	Exposed 46%	25%
rs199659271	C219R	192	Buried 5%	11%
*4IBM*				
rs121913156	R1201Q	1174	Intermediate 10%	0%
rs52800171	W1220L	1193	Buried 1%	0%
rs199580495	S1033F	1006	Intermediate 30%	0%
rs201979105	S1221A	1194	Buried 5%	0%

**Table 6:** RMSD and total energy after energy minimization of native structures and their mutant 3D models.

Molecules	RMSD (Å)	Total energy after energy minimization (kJ/mol)
*2HR7 native-type structure*	6.019	−22087.6969
2HR7 mutant (rs52836744)	6.007	−21968.7347
2HR7 mutant (rs121913145)	5.985	−21815.0585
2HR7 mutant (rs1051691)	5.997	−22160.7304
2HR7 mutant (rs55816055)	5.957	−21903.7516
2HR7 mutant (rs78827745)	5.991	−21816.4353
2HR7 mutant (rs79312957)	6.025	−21962.4576
2HR7 mutant (rs140762552)	5.992	−21823.0448
2HR7 mutant (rs141484557)	5.995	−22076.7362
2HR7 mutant (rs199659271)	5.98	−21736.5417

*4IBM native-type structure*	0.404	−13041.4646
4IBM mutant (rs121913156)	0.436	−13091.3512
4IBM mutant (rs52800171)	0.39	−12830.5659
4IBM mutant (rs199580495)	0.402	−11940.1628
4IBM mutant (rs201979105)	0.376	−13076.6808

**Table 7:** Protein stability upon mutation.

Molecules	Mutation	PDB residue number	RSA (%)	Predicted ΔΔ*G*	Outcome
*2HR7 native-type structure*					
2HR7 mutant (rs52836744)	G58R	31	33.2	−1.11	Destabilizing
2HR7 mutant (rs121913145)	H236R	209	30.5	−0.248	Destabilizing
**2HR7 mutant (rs1051691)**	**I448T**	**421**	**0**	**−2.403**	**Highly Destabilizing**
2HR7 mutant (rs55816055)	S353P	326	15.5	−0.339	Destabilizing
2HR7 mutant (rs78827745)	M65K	38	0	−1.568	Destabilizing
2HR7 mutant (rs79312957)	R413C	386	70.2	−0.931	Destabilizing
2HR7 mutant (rs140762552)	T107M	80	2.3	−0.387	Destabilizing
2HR7 mutant (rs141484557)	G262S	235	70.3	−0.775	Destabilizing
2HR7 mutant (rs199659271)	C219R	192	3.2	−0.039	Destabilizing
*4IBM native-type structure*					
4IBM mutant (rs121913156)	R1201Q	1174	11.5	−1.347	Destabilizing
**4IBM mutant (rs52800171)**	**W1220L**	**1193**	**2.1**	**−3.223**	**Highly Destabilizing**
4IBM mutant (rs199580495)	S1033F	1006	16.7	−0.888	Destabilizing
4IBM mutant (rs201979105)	S1221A	1194	9.5	−1.858	Destabilizing
